# Severe Necrotizing Pneumonia in Children, Houston, Texas, USA

**DOI:** 10.3201/eid1510.090589

**Published:** 2009-10

**Authors:** Anupama S. Kalaskar, Gloria P. Heresi, Audrey Wanger, James R. Murphy, Susan H. Wootton

**Affiliations:** University of Texas Health Science Center at Houston, Houston, Texas, USA

**Keywords:** Streptococcus pneumoniae, serotype 19A, necrotizing pneumonia, empyema, Texas, United States, bacteria, streptococci, letter

**To the Editor**: Routine vaccination of children with the 7-valent pneumococcal conjugate vaccine (PCV-7; Wyeth Pharmaceuticals, Collegeville, PA, USA), initiated in the United States in 2000, was followed within 2 years by an extensive and rapid decline in invasive pneumococcal disease (IPD) (*1*). During the past few years, increasing frequency of invasive disease including necrotizing pneumonia caused by serotypes not included in the vaccine has been reported (*2*). We show an expanded pattern of the changing spectrum of the disease associated with nonvaccine serotypes through this report of 4 cases of necrotizing pneumonia in children, caused by *Streptococcus pneumoniae* serotype 19A.

Over a 6-month period ending in March 2008, 4 children (median age 3.6 years, 1 with asthma) (Table) were brought to our hospital with signs of respiratory distress and a 4- to 7-day history of fever and cough. All had decreased breath sounds or crackles, and radiologic studies showed evidence of complicated pneumonia, which led to hospital admission (3 to an intensive care unit [ICU]). *S. pneumoniae* 19A was isolated from normally sterile sites with each child. All received intravenous antimicrobial drugs followed by an oral antimicrobial drug regimen and were discharged in good health. By reviewing immunization records, we confirmed that all had completed the PCV-7 series before becoming ill.

**Table:** Characteristics of patients in study of severe necrotizing pneumonia in children, Houston, Texas, USA, 2007–2008*

Characteristic	Patient			
	1	2	3	4
Age, y	4.1	2.8	3.4	3.7
Race	Black	White	Black	Black
Sex	M	M	F	F
Completed PCV-7 series	Yes	Yes	Yes	Yes
Co-illnesses	Asthma	None	None	None
Clinical signs				
Date	2007 Nov	2007 Nov	2007 Dec	2008 Feb
Temperature, °C	37.6	**38.9**	37.9	**38.7**
Pulse, beats/min	**154**	**160**	**162**	**188**
Respiratory rate, breaths/min	**70**	**60**	**40**	**42**
Blood pressure, mm Hg	95/53	**122/84**	**110/78**	106/69
Oxygen saturation on room air, %	**85**	**90**	100	**86**
Symptoms/signs (hospital)	Fever, cough, dyspnea, headache, abdominal pain, decreased activity	Fever, cough, dyspnea, congestion	Fever, cough	Fever, cough, decreased appetite
Hospital course				
Site pathogen isolated	Blood and pleural fluid	Blood and pleural fluid	Pleural fluid	Blood
IV antimicrobial drugs, d	21	9	14	33
IV antimicrobial drugs (MIC, μg/mL)	Cefotaxime (0.06)	Vancomycin (0.75); ceftriaxone (0.19)	Vancomycin (1); resistant to cephalosporin	Vancomycin (0.50); ceftriaxone (0.064)
VATS† (duration, d)	Yes (2)	Yes (2)	Yes (2)	No
ICU stay	Yes	Yes	No	Yes
Intubated (duration, d)	Yes (5)	Yes (5)	No	Yes (22)
Duration of hospitalization, d	22	11	15	28
Oral antimicrobial drugs after discharge (duration, d)	Cefdinir (7)	Cefdinir (14)	Linezolid (14)	Cefdinir (7)

*PCV, pneumococcal conjugate vaccine; IV, intravenous; VATS, video-assisted thoracoscopic surgery; ICU, intensive care unit. **Boldface** indicates clinically significant differences. †Hospitalization day that VATS was performed.

During the same period, complicated pneumonia was identified in 7 other inpatients by using the International Classification of Diseases, 9th revision, codes for necrotizing pneumonia and empyema and Current Procedural Terminology codes for thoracoscopic surgery (median age 4.3 years); 2 had asthma, 1 had congenital diaphragmatic hernia with resultant left lung hypoplasia. No causative organism was identified for any of these cases.

As illustrated by our 4 cases, serotype 19A is emerging as an increasing cause of severe disease such as complicated pneumonia. Although the incidence of IPD in general has decreased since the introduction of PCV-7, the emergence of nonvaccine serotypes as a cause of severe disease is becoming more prevalent. Among 8 geographic areas in the United States, the incidence of IPD caused by nonvaccine serotypes in children <5 years of age increased from 16.3 cases/100,000 population to 19.9 cases/100,000 population, respectively, from prevaccine years 1998–1999 to in 2004 (*3*).

Because organisms were not isolated from the 7 other patients with necrotizing pneumonia during the same period, we are unable to comment on whether *S. pneumoniae* 19A was the predominant cause of necrotizing pneumonia in our study. However, in comparing our patients with these 7 patients, those with *S. pneumoniae* 19A appear to have had a more complicated course of illness, longer hospital stays (mean 19 days vs. 13 days), and a longer course of intravenous antimicrobial drugs (mean 19.2 days vs. 17 days). Although these 7 patients required more video-assisted thoracoscopic surgery than did our 4 patients (100% vs. 75%), those with *S. pneumoniae* 19A necrotizing pneumonia had a more severe clinical course of illness resulting in more ICU admissions (75% vs. 29%) and intubations (75% vs. 14%).

All 4 of our patients had completed the PCV-7 series before becoming ill. The emergence of nonvaccine serotypes as a cause of severe disease may be caused by serotype replacement and increased nasopharyngeal carriage of nonvaccine serotypes after receiving PCV-7 (*4*). Our report supports the theory of serotype replacement. The increasing incidence of invasive disease caused by nonvaccine serotypes has prompted development of an expanded pneumococcal vaccine to include serotypes 1, 3, 5, 6A, 7F, and 19A in addition to those covered by PCV-7 (*5*). The need for this expanded vaccine is becoming more evident as the number of children with severe pneumococcal disease increases due to current nonvaccine serotypes.
